# Early Menarche is a Risk Factor for Short Stature in Young Korean Females: An Epidemiologic Study

**DOI:** 10.4274/jcrpe.galenos.2018.2018.0274

**Published:** 2019-09-03

**Authors:** Sol Kang, Yoon Mo Kim, Jun Ah Lee, Dong Ho Kim, Jung Sub Lim

**Affiliations:** 1 Korea Cancer Center Hospital, Department of Pediatrics, Seoul, Republic of Korea

**Keywords:** Early menarche, short stature, adult height, obesity, KNHANES

## Abstract

**Objective:**

To assess the association between age at menarche and adult height [and body mass index (BMI)] in young Korean females and also to investigate whether early menarche (<12 years) is a risk factor for short stature and obesity in young Korean females.

**Methods:**

Data on 1148 females aged 18-30 years and 612 mother (612 pairs of mothers and daughters) from the 6^th^ Korea National Health and Nutrition Examination Survey (2013-2015) were analyzed.

**Results:**

Among 1148 females, 256 (22.3%) had early menarche. Their stature was approximately 0.445 cm shorter when menarche had occurred one year earlier. The prevalence of short stature (≤153 cm) and obesity (BMI ≥25) was higher in females with early menarche compared to those with later menarche (short stature: 10.5% vs 6.4%, obesity; 20.7% vs 13.1%, all p<0.001). In multivariate regression, the odds ratio (OR) for short stature was 2.62 [95% confidence interval (CI): 1.26-5.44] after adjusting for current age and mother’s height. OR for obesity was 1.74 (95% CI: 0.98-3.07) after adjusting for age and maternal BMI.

**Conclusion:**

Final height in girls is influenced by age of menarche. Early menarche increased the risk for adult short stature in young Korean females.

**What is already known on this topic?**

In European countries, age at menarche was positively associated with final adult height and negatively associated with body mass index.

**What this study adds?**

This is the first study to show that early menarche is a risk factor for short adult stature in Korean females. The odds ratio for short stature in females with early menarche was 2.62 after adjusting for mother’s height.

## Introduction

Age of menarche is known to be influenced by several factors, such as genetics, ethnicity, geography, socioeconomic status and especially nutritional status (1,2,3,4). Several studies including this one have reported a relationship between early menarche age (<12 years) and the risk of obesity, insulin resistance, metabolic syndrome, nonalcoholic fatty liver disease, diabetes and cardiovascular disease in adulthood (5,6,7,8,9). Thus, management of risk factors for early menarche in the pediatric population may reduce the risk of adult metabolic disease.

Short stature is typically defined as an adult height that is more than two standard deviations (SD) below the mean for age and sex (10). In developed countries, this typically includes adult men who are shorter than 166 cm and adult women who are shorter than 153 cm. Several factors might cause short adult height. The main factors appear to be the effects of multiple familial genes and environment, and the complex interplay between these. In addition, short adult height can be caused by pathological states, including genetic disease such as Turner syndrome, prolonged chronic disease, malnutrition, prolonged treatment with certain drugs (steroids) and hormone deficiency states such as growth hormone deficiency. Short adult height may also occur because of early fusion of growth plates as a result of precocious puberty (11).

In Europe and the USA, women with earlier menarche were reported to have reached shorter adult height compared to women who had menarche at a later age (12,13,14). Furthermore, in Asian countries including Korea, female age of menarche has recently shown a downward trend to younger age (5,15,16).

The aim of this study was to investigate whether final adult height is associated with age at menarche in young Korean females. We also assessed whether an independent association exists between early menarche and short adult stature or obesity in Korean females. For this purpose, we investigated the data of 12,537 women who participated in the Korea National Health and Nutrition Examination Survey (KNHANES-VI).

## Methods

The data of the 6^th^ KNHANES-VI (2013-2015) data were used in the study. KNHANES-VI is a cross-sectional survey with multi-staged, stratified sampling design and offers nationally representative data conducted by the Division of Chronic Disease Surveillance, Korea Centers for Disease Control and Prevention (17). Written informed consent was secured by all of the participants before the study had begun, and the KNHANES was conducted following ethical approval by the Institutional Review Board of the Korea Centre for Disease Control and Prevention (No: 2013-07CON-03-4C, 2013-12EXP-03-5C).

Among the 12,537 females who participated in KNHANES-VI, we selected data on menarcheal age and anthropometric variables. There were 1148 young females aged 18 to 30 years and mothers’ height and weight data were available in 612 of the 1148 subjects. Weight was determined to the nearest 0.1 kg on a medical balance (GL-6000-20, CAS, Seoul, Korea) and height was measured to the nearest 0.1 cm with a wall-mounted stadiometer (Seca 220, Seca, Hamburg, Germany). Body mass index (BMI) was calculated by dividing the weight by the height squared (kg/m^2^). Height in Korean females reaches a near plateau at age 16 according to 2017 Korean National Growth Charts (18).

“Age of menarche” is defined as age of the first menstrual period and the data was collected using the questionnaire method. The question was open-ended: “At what age did you have your first menstrual period (menarche)?” Age of years represents age between 11.00-11.99 years. We defined early menarche as <12 years. Short stature was defined as a height less than ≤153 cm (≤5^th^ percentile of a female Korean population) and obesity as a BMI ≥25, using Asian criteria (19). Household income as a surrogate marker of socioeconomic status was assessed according to the following categorical variables: low (1Q), lower middle (2Q), upper middle (3Q), and high (4Q) in KNHANES.

### Statistical Analysis

Data related to anthropometric measurements and other covariates were stratified by early menarche and later menarche. The Student’s t-test and chi-square test were used in the comparison of early menarche and later menarche. Continuous variables are reported as means±SD, and categorical variables are reported as percentages (%). Linear regression analysis was used to evaluate the predictors of the subject’s height as a dependent variable using heights at menarche as predictive variables, controlling for current age. For the assessment of odds ratios (ORs) of short stature or obesity according to early menarche, multivariable logistic regression was used. The ORs including 95% confidence interval (CI), between early menarche and short stature (or obesity) were calculated before and after adjusting for age, and other confounders. In the final analysis, household income was excluded as there was no significant difference of prevalence of short stature from quartile to quartile. All statistical analyses were performed by using SPSS 17.0 for Windows (SPSS Inc., Chicago, IL, USA). P values <0.05 were considered significant.

## Results

The characteristics of the study subjects were divided according to early menarche or later menarche and are summarized in Table 1. At the time of the study, the mean±SD current age of all subjects was 23.5±3.5 years. Mean±SD age of menarche was 12.7±1.6 years. Mean±SD (range) height was 161.6±5.8 (138-179) cm and mean±SD (range) BMI was 21.6±3.7 (15-49) kg/m^2^.

**Figure 1:**
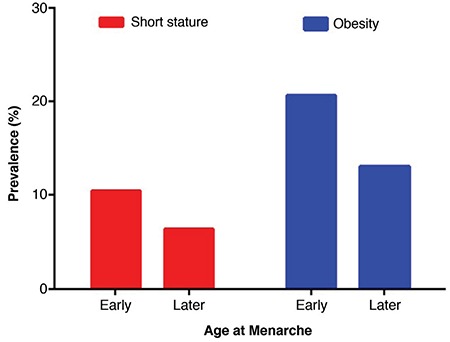
Prevalence of short stature and obesity according to early and later menarche. Females with early menarche had higher prevalence of short stature (10.5 vs 6.4%) and obesity (20.7 vs 13.1%)

Among the 1148 female subjects, 256 (22.3%) had early menarche and 892 had later menarche. Mean±SD current age was significantly younger in the early menarche group (22.9±3.4 *vs* 23.7±3.5 years; p=0.001). This group was also significantly shorter (160.4±5.1 *vs* 161.9±6.0 cm; p<0.001) and had a higher BMI (22.4±3.8 *vs* 21.3±3.5; p<0.001) than the later menarche group, The early menarche group also had a higher prevalence of short stature (10.5% *vs* 6.4%) and obesity (20.7% *vs* 13.1%) (see Figure 1). However, there was no difference in prevalence affected by household income.

In the subgroup of subjects with available maternal anthropometric data (n=612), the mean±SD age of the mothers was 50.3±4.6 years and maternal mean±SD age at menarche was 14.3±1.7 years. Differences in age, age at menarche, height and BMI between mother and daughter were 27.5±3.5 years, 1.5±2.0 (-4 to 8) years, 4.4±5.9 (-20 to 23) cm and 2.5±3.9 kg/m^2^, respectively.

There was no difference in mother’s age, height, weight and BMI values between the early menarche and later menarche groups. However, the mothers of subjects with early menarche had earlier menarche than mothers of subjects with later menarche (13.8±1.5 *vs* 14.4±1.7 years; p<0.001). There was also no difference in prevalence of short stature and obesity in the mothers.

**Figure 2:**
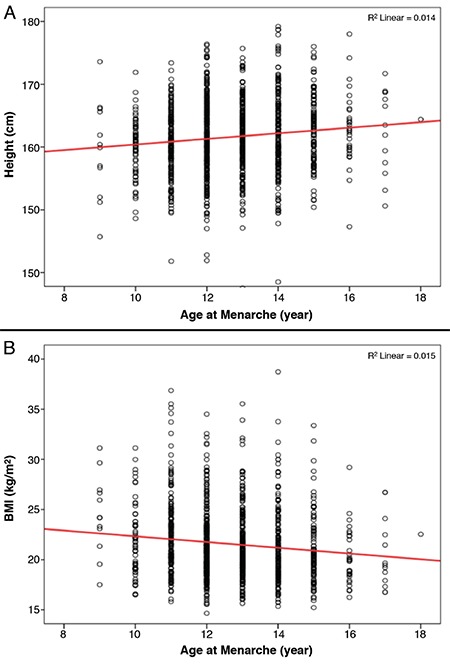
A, B) Height and body mass index according to age at menarche. Females were 0.445 cm shorter when menarche occurred 1 year earlier

Height and BMI according to age at menarche are depicted in Figure 2. In linear regression, female grew approximately 0.445 cm shorter when menarche occurred one year earlier calculated as: subject’s height (cm)=subject’s age at menarche (years) x 0.445 - subject’s age (years) x 0.03+156.56 (R^2^=0.014; p<0.001). The ORs for short stature and obesity in females with early menarche compared to later menarche are summarized in Table 2. The crude OR for short stature in a female with early menarche was 1.73. The OR decreased to 1.71 after adjusting for current age (Model 1) and increased to 2.62 after further adjusting for mother’s height (Model 2). Here, the Exp(B) of mother’s height was 0.799 (95% CI: 0.742-0.860). The OR for obesity in females with early menarche was 1.73. The OR increased to 1.79 after adjusting for current age (Model 1) and subsequently decreased to 1.74 (95% CI: 0.98-3.07) after adjusting for age and mother’ BMI (Model 2).

## Discussion

In this study, we found that for each year earlier that menarche occurred in young Korean females final height was 0.445 cm less. We also found that females with early menarche had a 10.5% chance of having short adult stature, the rate of which was 2.62-fold higher than those with later menarche. In addition, the OR for obesity in females with early menarche was 1.73 compared to those with later menarche.

The prevalence of early menarche has increased dramatically in Korea based on our study (5). In this study, the percentage of subjects who experienced menarche before the age of 12 years was 22.3%, while this percentage in the mothers of the subjects was 2.5%. The prevalence in the subjects was nine times higher than that of their mothers. Similar to many other countries, South Korea has shown rapid decrease of female age in menarche from mid-20th century to the present, and this is probably due to improvements in nutrition and living conditions (13,20). In our previous study, we reported the rapid decrease of mean age of menarche over time, from 15.62±1.88 years for females born between 1950 and 1954, to 13.11±1.52 years for those born between 1980 and 1984, to 12.60±1.14 years for those born between 1990 and 1994 (5). This trend might be due to changes in the socioeconomic environment, the most important of which is probably improvement in nutritional status (13). The nutritional status of South Korea rapidly improved after the Korean Civil War. South Koreans rapidly accepted western culture, especially in terms of dietary habits, after the 1988 Olympic Games, while North Korean refugees still show an age of menarche around 16.0±2.1 years (20). Nutrition, in particular, appears to play an important role in the time of onset of menarche. There are great many reports indicating that girls with higher body weight, higher BMI and more body fat reach menarche at an earlier age (13,21,22). It has been suggested that a ‘critical weight’ is needed for menarche to occur (23,24). Furthermore, other factors such as birth weight, prenatal nutrition, type of diet and exposure to endocrine disruptors, have been suggested as likely contributory factors for earlier pubertal development and early menarche (25,26,27). Another suggested reason for the increasing prevalence of early menarche in Korean girls is a rapid increase in the prevalence of central precocious puberty (CPP). CPP can cause early menarche in girls and result in short adult stature due to early epiphyseal fusion (11). The annual incidence of CPP in girls has significantly increased from 3.3 to 50.4 per 100,000 girls during 2004 to 2010 in Korea (28). The incidence of girls diagnosed with CPP has markedly increased in the 21st century. In Denmark for instance, it is much higher compared to 40 years ago (29).

In this study, 10.5% of females with early menarche had a short stature. The OR for short stature was 2.62-fold after adjusting for current age and mother’s height in females with early menarche compared to those with later menarche. In basic terms, for each year of delay in age at menarche, a Korean female will grow to be be approximately 0.445 cm taller in her final height. This finding agrees studies from other countries (12,21,30,31). According to the European Prospective Investigation into Cancer and Nutrition study, based on 286,205 women from nine European countries, women grew approximately 0.35 cm taller when menarche occurred one year later (range by country: 0.13-0.50 cm) (12). Furthermore, a 1-year increase in age of menarche caused an increase in standing height, leg length and trunk height of 0.76, 0.41 and 0.35 cm, respectively, in a USA birth cohort (31).

The pathogenesis of this trend in age of menarche may be explained by the earlier closure of epiphyseal growth plates due to an increase in ovarian estrogens (32,33). A low dose of estrogen will induce stimulation of the growth hormone-insulin-like growth factor 1 axis and a pubertal growth spurt in early puberty. However, increase in estrogen binding to its receptors in the growth plate cartilage might cause early epiphyseal fusion by advancing growth plate senescence (33). A delay in menarche allows continued growth of long bones before epiphyseal fusion, leading to an increase in adult height. Gonadotropin releasing hormone analog (GnRHa) treatment in girls with CPP could improve final adult height and increase the age of menarche close to that of the general population. The height gain achieved after GnRHa treatment in children with CPP, depends on the age of onset of puberty and onset of treatment (34).

In this study, the OR for obesity in females with early menarche was 1.73. However, after adjusting for age and maternal BMI, we failed to find an association between early menarche and obesity (p=0.058). The most plausible reason for this might be the relatively small number of subjects analyzed. Additionally, socioeconomic factors assessed in our study failed to demonstrate an association with adult short height. This finding agrees with a previous Korean study and supports the hypothesis that socioeconomic status is not an independent predictor of age at menarche or final height in well-developed countries (35,36).

### Study Limitations

The major strength of the present study is that it is based on a national representative study population. However, this study has some limitations. First, the cross-sectional nature of the study prohibits making conclusions regarding the existence of a causal relationship between age of menarche and adult height. Second, we could not adjust for other confounders such as the fathers’ height and birth weight, which might influence the subjects’ adult height. Recent studies showed an association between lower birth weight and early menarche (26). Finally, the age at menarche was self-reported. It is known that age at menarche based on recall data is not very accurate, especially when the time between menarche and current age is more than three years (37). However, other studies have shown high correlations (R=0.67 to 0.79) between age at menarche by recall during middle-age and the original childhood data (38).

## Conclusion

In conclusion, we found that early menarche is a risk factor for shorter adult stature and obesity in young Korean females. To the best of our knowledge, this is the first study to show that early menarche is a risk factor of short adult stature in Korean females. Some girls, despite being of normal height during childhood and during the pubertal years might grow to short adult stature due to earlier fusion of the growth plates. In light of the rapidly increasing prevalence of early menarche, knowledge concerning onset of puberty, progression tempo and age at menarche is important in identifying females at risk. Further long term cohort investigations are needed to fully explain these causal relationships.

## Figures and Tables

**Table 1:**
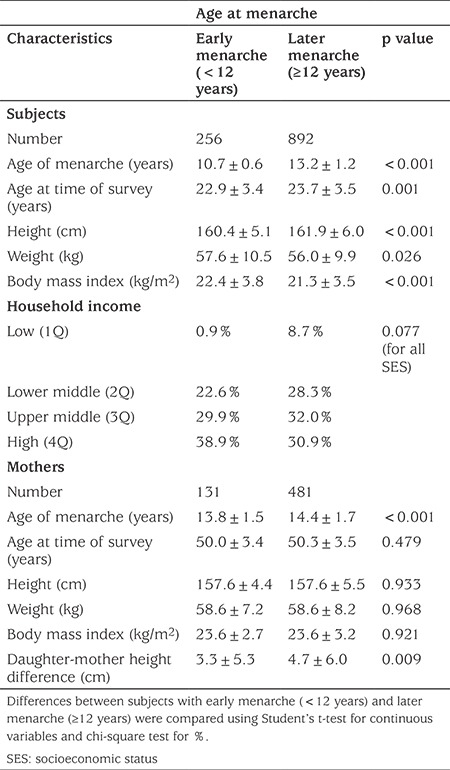
Characteristics of the subjects stratified by age at menarche

**Table 2:**
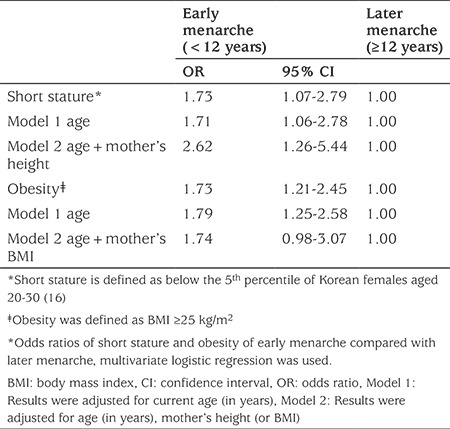
Odds ratios for short stature and obesity between subjects with early menarche and the reference group
